# Genes associated with diagnosis and prognosis of Burkitt lymphoma

**DOI:** 10.1049/syb2.12054

**Published:** 2022-11-10

**Authors:** Albert Doughan, Samson Pandam Salifu

**Affiliations:** ^1^ Department of Biochemistry and Biotechnology College of Science Faculty of Biosciences Kwame Nkrumah University of Science and Technology (KNUST) Kumasi Ghana; ^2^ Kumasi Centre for Collaborative Research in Tropical Medicine (KCCR) Kumasi Ghana

**Keywords:** Africa, biomarker, Burkitt lymphoma, differential expression analyses, hub genes

## Abstract

Burkitt lymphoma (BL) is one of the most aggressive forms of non‐Hodgkin's lymphomas that affect children and young adults. The expression of genes and other molecular markers during carcinogenesis can be the basis for diagnosis, prognosis and the design of new and effective drugs for the management of cancers. The aim of this study was to identify genes that can serve as prognostic and therapeutic targets for BL. We analysed RNA‐seq data of BL transcriptome sequencing projects in Africa using standard RNA‐seq analyses pipeline. We performed pathway enrichment analyses, protein–protein interaction networks, gene co‐expression and survival analyses. Gene and pathway enrichment analyses showed that the differentially expressed genes are involved in tube development, signalling receptor binding, viral protein interaction, cell migration, external stimuli response, serine hydrolase activity and PI3K‐Akt signalling pathway. Protein–protein interaction network analyses revealed the genes to be highly interconnected, whereas module analyses revealed 25 genes to possess the highest interaction score. Overall survival analyses delineated six genes (ADAMTSL4, SEMA5B, ADAMTS15, THBS2, SPON1 and THBS1) that can serve as biomarkers for prognosis for BL management.

## Graphical abstract

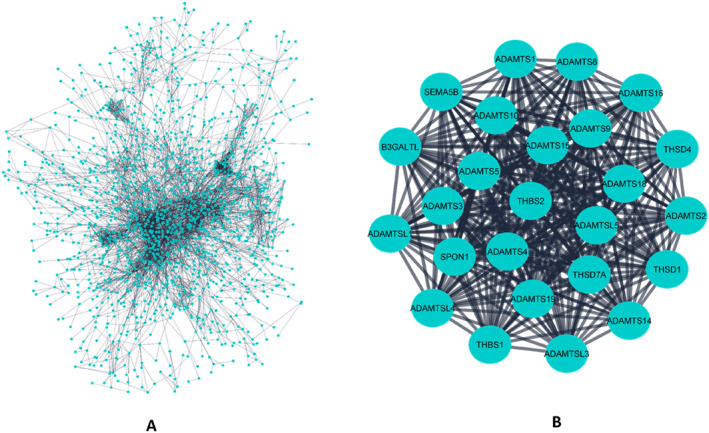

Analysing gene expression data of Burkitt lymphoma transcriptome sequencing from African patients revealed six biomarkers (ADAMTSL4, SEMA5B, ADAMTS15, THBS2, SPON1 and THBS1) as possible indicators for diagnostic and prognostic targets towards BL management.

## 1 INTRODUCTION

Burkitt lymphoma (BL) is one of the most aggressive forms of non‐Hodgkin's lymphomas that affect children and young adults. Overall, BL accounts for 1%–5% of all non‐Hodgkin's lymphomas and clinically presents as a conspicuous accumulation of tissues in the cheek and jaws of its victims [1]. The pattern and distribution of BL vary depending on age, sex and geographical location, suggesting the roles of both genetic and environmental factors in the development of the disease. BL is more common in males than in females with a ratio of 4:1 [2], accounts for 36% of all childhood cancers (2–16 years), 70% of childhood lymphomas and 5% of lymphomas for both adults and children [3]. In equatorial Africa, BL is usually associated with *Plasmodium falciparum* and Epstein‐Barr virus infections, where the latter is seen in 90% of BL cases [4]. Although BL can be treated if detected early, identifying reliable biomarkers has the added advantage of outcome assessment.

Identifying biomarkers associated with BL is important as it gives detailed information about the likely outcome of a treatment regimen. Panea et al. [5] identified *BCL7A* and *BCL6* as potential predictive biomarkers in a Kenyan BL cohort. Kaymaz et al. [6] also found four members of the Proteasome 20S Subunit Beta (*PSMB9*, *PSMB10*, *PSMB8* and *PSMB2*) to be associated with BL progression. The signature translocation event in BL involving the *MYC* and its deregulation has also been revealed by Kaymaz et al. [6], Abate et al. [7] and Panea et al. [5] through RNA‐seq analyses. *LEF1* has been reported as a biomarker for assessing prognosis in multiple human cancers, including BL [8]. However, the following gaps persist in the current BL biomarkers; [1] there is no established consensus regarding the identified biomarkers implicated in BL progression across the various geographic locations; [2] there is limited information about the interactions among the identified biomarkers; and [3] there is inadequate information on how the biomarkers compare with biomarkers from other human cancers. The present study leverages these gaps by analysing a pool of RNA‐seq data from different geographical locations in Africa. This work provides a broader picture of the genetic landscape of BL in Africa, and the identified biomarkers could serve as a more representative biomarker associated with BL progression. Ultimately, the biomarkers could be further explored as potential targets for the diagnostic and prognostic makers for management of BL.

## 2 MATERIALS AND METHODS

### 2.1 Data acquisition

**FIGURE 1:**
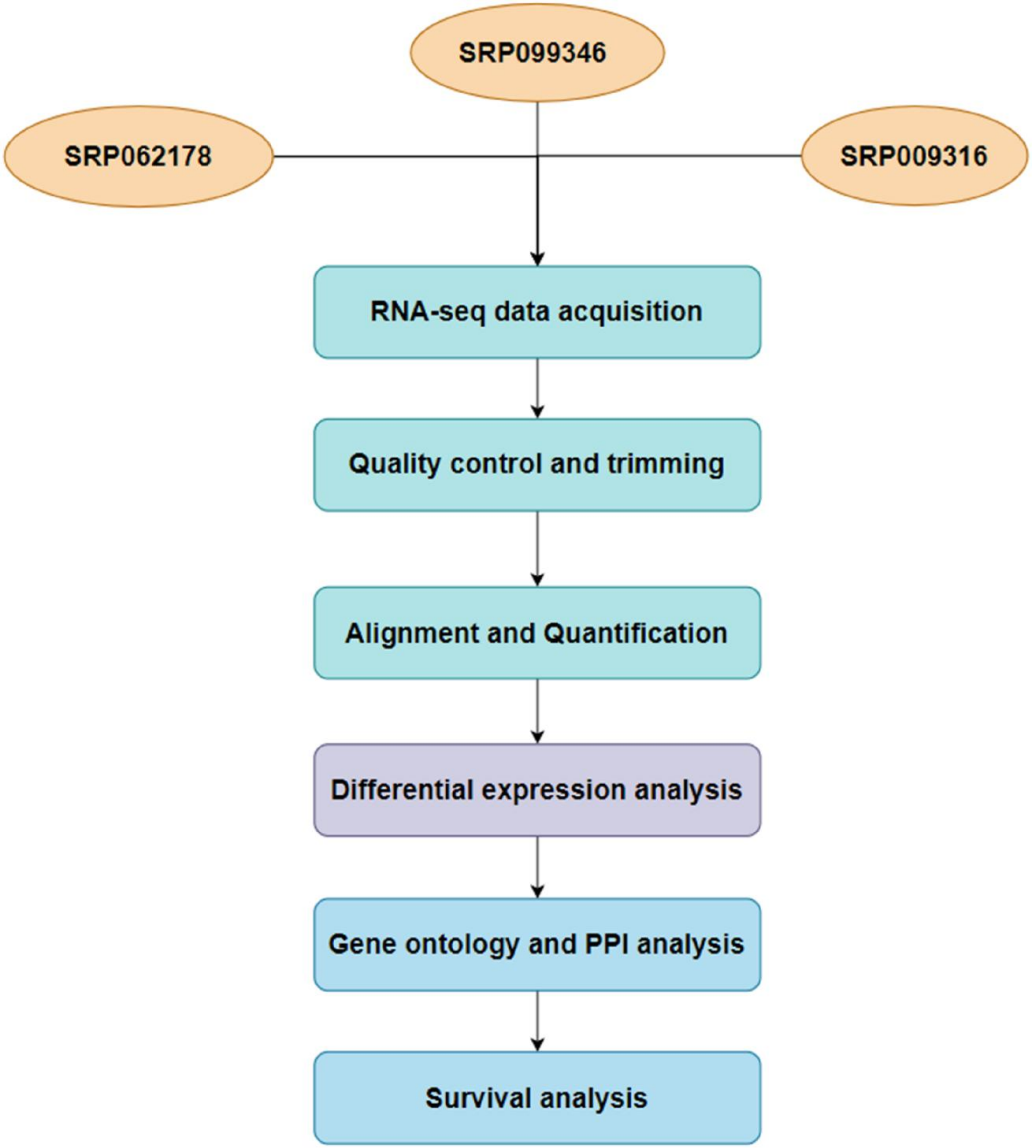
A diagrammatic representation of the various analyses performed in this study.

In this study, we downloaded and analysed RNA‐seq data obtained from National Centre for Biotechnology Information Short Read Archive (NCBI‐SRA) that met our set criteria of [1] the data being generated from African patients with BL within the last 10 years, [2] diagnosis of BL being conducted and confirmed by two experienced oncology pathologists and [3] data is available as paired‐end sequences. We excluded patients with either immunodeficient BL or sporadic BL. With these criteria, we arrived at three different sets of RNA‐seq data generated by Abate et al. [7], Lombardo et al. [9] and Kaymaz et al. [6]. The raw RNA‐seq data was downloaded from the NCBI‐SRA under the Accession numbers SRP062178, SRP099346 and SRP009316. Overall, 100 RNA‐seq data were included in the analysis; 50 cases and 50 control groups. Figure 1 provides an overview of the steps undertaken in this study.

### 2.2 Data pre‐processing, trimming and alignment

The raw RNA‐seq datasets in the FASTQ format were evaluated for gastric cancer (GC)‐content, k‐mer distribution, sequence counts, quality scores, base quality, adaptor content and sequence duplication levels with FastQC *v0.11.8* [10]. MultiQC *v1.9* [11] was used to summarise all the reports generated by FastQC. Trimmomatic *v0.39* [12] was used to trim off adaptors, low‐quality bases and short sequences. Sequences that passed the quality trimming stage were mapped to the human reference genome (GRCh38) using STAR *v2.7.7a* [13]. featureCounts *v1.6.1* was used for gene quantification.

### 2.3 Differential expression analysis (DEA) and gene ontology (GO) analyses

Differential expression analysis (DEA) was performed within the R statistical tool's environment using DESeq2 under the default settings. Genes with adjusted *p*‐values less than 0.05 were considered as differentially expressed between the two conditions (cases vs. controls). Gene ontology (GO) analysis was performed using the ShinyGO online platform [14]. GO achieves high throughput annotation of genes based on the biological process, molecular function, cellular component and biological pathway analyses. Pathways and biological processes (BP) with adjusted *p* values less than 0.05 were considered statistically significant.

### 2.4 Protein–protein interaction (PPI) network

STRING [15] was used to retrieve the complex interactions between the genes and visualised with Cytoscape [16]. MCODE [17], a Cytoscape‐based software that topologically clusters a network to locate areas of dense connectedness was used to identify hub genes.

### 2.5 Hub genes expression in tumours and survival analysis

The expression of the hub genes in other human cancers was assessed using the Gene Expression Profiling Interactive Analysis 2 (GEPIA2) online tool [18]. GEPIA2 is an interactive resource that enables researchers to gather valuable information from genes using gene expression data from The Cancer Genome Atlas (TCGA) and Genotype‐Tissue Expression (GTEx) projects. Patient survival analysis was performed using the GEPIA2.

## 3 RESULTS

### 3.1 Selecting differentially expressed genes

**FIGURE 2:**
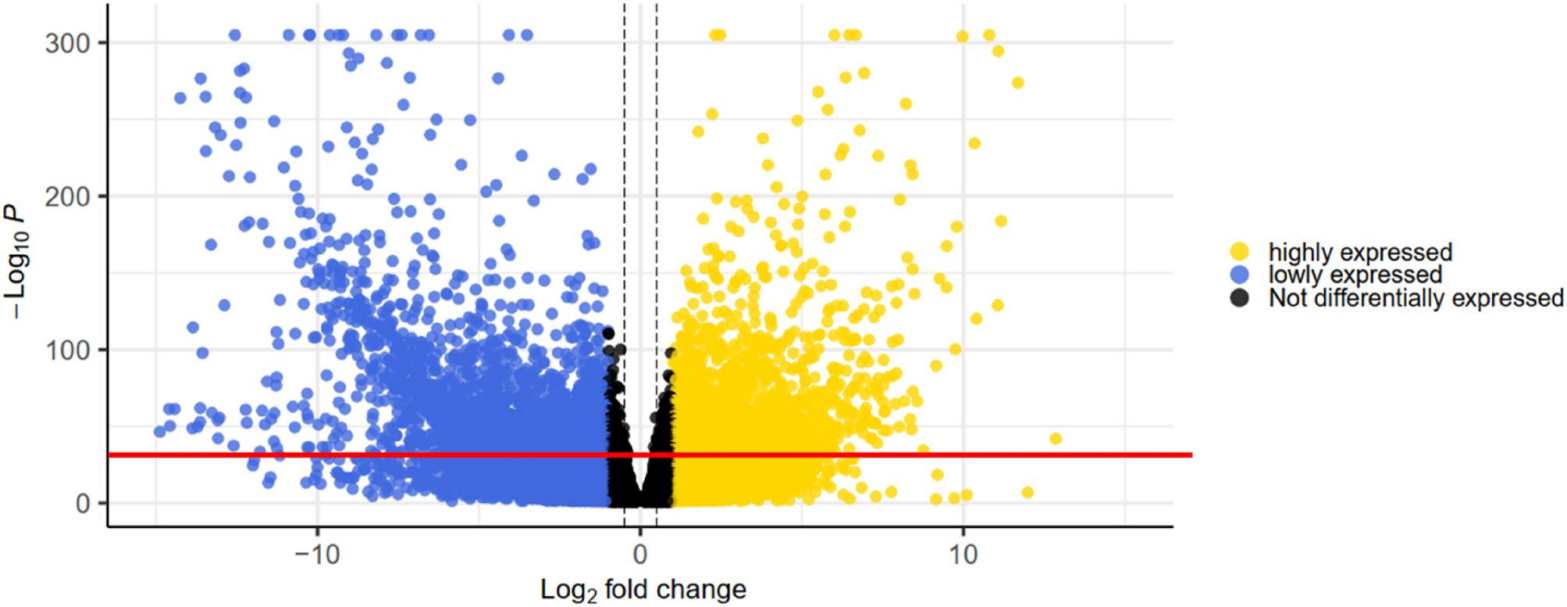
A volcano plot of the RNA seq results. In all 6314 differentially expressed genes were determined. The blue and yellow dots represent down‐regulated and up‐regulated genes respectively. The black dots are not differentially expressed genes, and all dots below the red line are non‐significant genes.

In the present study, we analysed 100 RNA‐seq data generated from patients with BL and geographically matched healthy controls. Compared with the normal samples, 21,286 genes were differentially expressed (Figure 2). Setting the fold change threshold to ±2, a total of 6314 genes were arrived at, of which 4291 and 2023 were down‐ and up‐regulated respectively.

### 3.2 Gene ontology (GO) and pathway enrichment analyses

**FIGURE 3:**
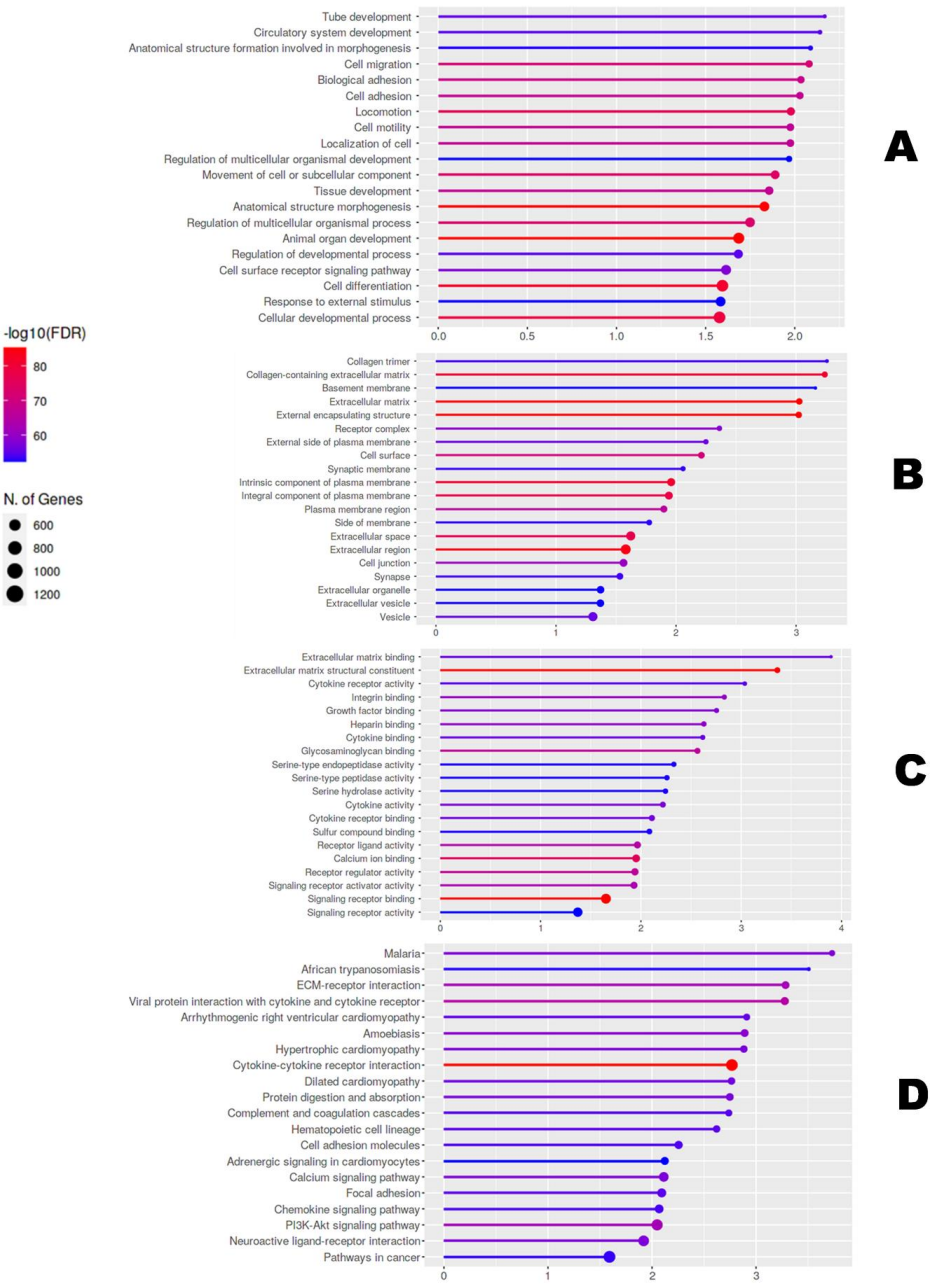
Gene ontology (GO) and pathway enrichment analysis of the DEGs. (a) GO biological process, (b) GO cellular component, (c) GO molecular function and (d) Kyoto Encyclopaedia of Genes and Genomes (KEGG) pathway. The dot size represents the number of DEGs implicated in the respective GO term. The colour intensity represents the significance level; red is more significant than blue.

We used the ShinyGO online tool to establish the GO classifications and pathways for the detected DEGs to elucidate their functions. The GO terms were grouped as BP, molecular functions (MF) and cellular components (CC), together with the Kyoto Encyclopaedia of Genes and Genomes (KEGG) was used to map the molecular pathways (Figure 3). The results showed that the DEGs were primarily enriched in BP, such as tube development (4.1 × 10^−56^), circulatory system development (1.7 × 10^−55^), anatomical structure formation involved in morphogenesis (2 × 10^−53^), cell migration (1.8 × 10^−72^) and biological adhesion (2 × 10^−69^). The DEGs for MF were enriched in signalling receptor binding (3.8 × 10^−32^), extracellular matrix binding (6.5 × 10^−14^), extracellular matrix structural constituent (3.8 × 10^−32^), cytokine receptor activity (2.8 × 10^−13^), integrin binding (1.9 × 10^−16^) etc. The results from the CC analysis showed that the genes are involved in collagen trimer (3.8 × 10^−17^), collagen‐containing extracellular matrix (1.9 × 10^−73^), extracellular matrix (1.6 × 10^−82^), external encapsulating structure (1.6 × 10^−82^), receptor complex (6.5 × 10^−28^) etc. The DEGs were significantly enriched in the following pathways; Malaria (3 × 10^−11^), ECM‐receptor interaction (2.0 × 10^−14^), viral protein interaction with cytokine and cytokine receptor (4.9 × 10^−16^), haematopoietic cell lineage (3.7 × 10^−9^), protein digestion and absorption (8.8 × 10^−11^) etc. (Figure 3).

### 3.3 PPI network analyses and module selection

**FIGURE 4:**
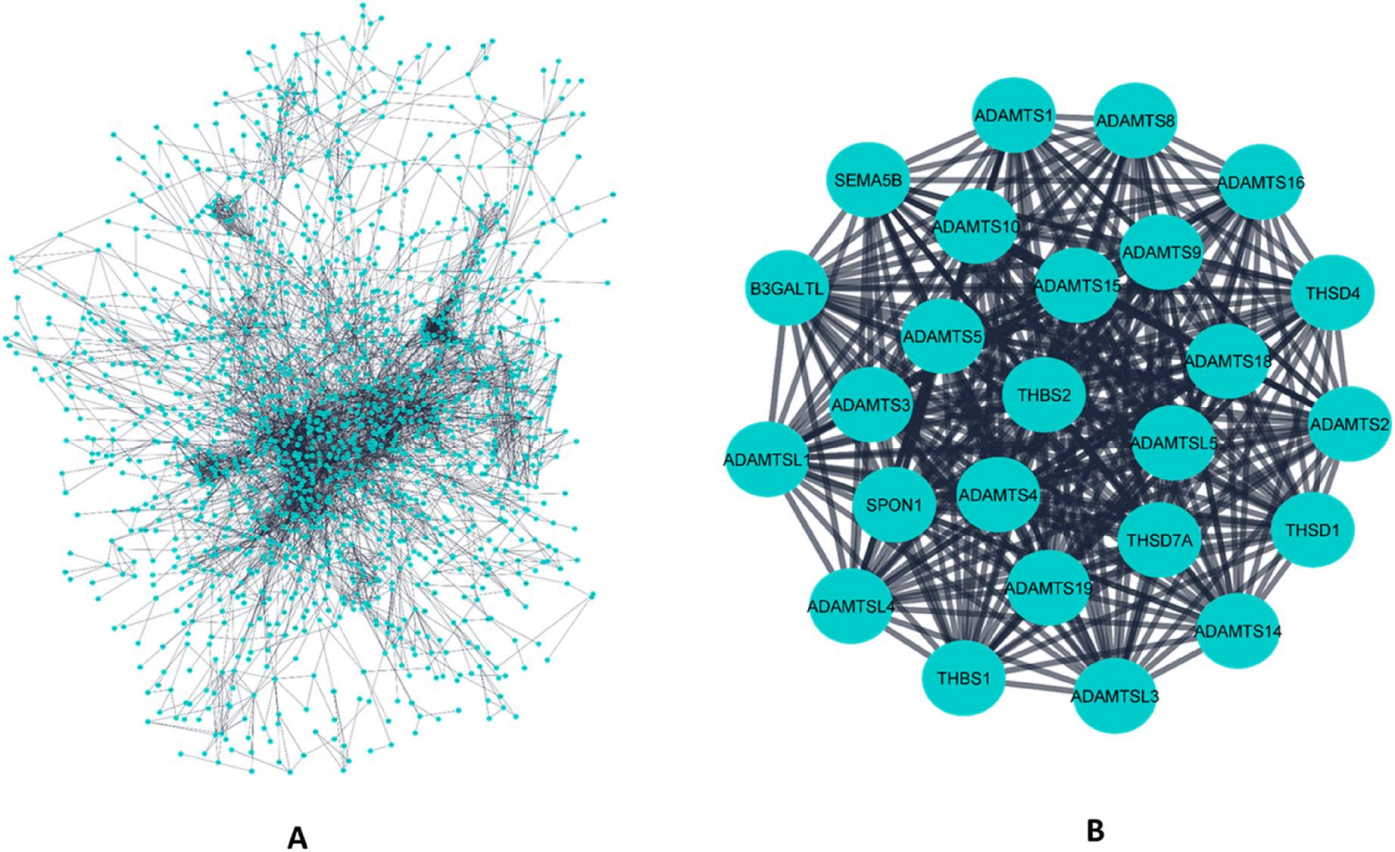
Protein–protein interaction (PPI) network of the differentially expressed genes. (a) Interactions between all the 6314 genes. (b) Interactions between the highly interconnected genes. Circles and dark lines represent genes and interactions respectively.

Cytoscape was used to generate the protein‐protein interaction (PPI) network plot of all the DEGs (Figure 4a). The most highly interconnected regions were selected using the MCODE plugin in Cytoscape. Based on the interaction score, the resulting PPI networks were ranked. Figure 3b shows the network with the highest interaction score (24.92) among all the 78 networks. It contained 25 nodes (genes) and 299 edges (interactions). Through GO and KEGG analysis, the 25 hub genes were found to be enriched in metalloendopeptidase activity, extracellular matrix organisation, extracellular matrix, Malaria etc. (Table 1).

**TABLE 1:** Gene ontology and KEGG enrichment analyses of the 25 hub genes

GO category	Term ID	Description	Adjusted *p*‐value	No. of genes
MF	GO:0004222	Metalloendopeptidase activity	5.74 × 10^−31^	17
MF	GO:0008237	Metallopeptidase activity	2.52 × 10^−26^	17
MF	GO:0004175	Endopeptidase activity	5.34 × 10^−20^	17
MF	GO:0008233	Peptidase activity	3.26 × 10^−17^	17
MF	GO:0016787	Hydrolase activity	6.32 × 10^−08^	17
BP	GO:0030198	Extracellular matrix organisation	6.17 × 10^−20^	17
BP	GO:0019538	Protein metabolic process	8.15 × 10^−4^	20
BP	GO:0045229	External encapsulating structure	6.98 × 10^−20^	17
BP	GO:0006508	Proteolysis	6.53 × 10^−12^	19
BP	GO:0016043	Cellular component organisation	7.5 × 10^−4^	21
CC	GO:0031012	Extracellular matrix	2.01 × 10^−24^	20
CC	GO:0030312	External encapsulating structure	2.09 × 10^−24^	20
CC	GO:0005576	Extracellular region	2.25 × 10^−10^	23
CC	GO:0071944	Cell periphery	3.14 × 10^−6^	22
CC	GO:0005788	Endoplasmic reticulum lumen	1.10 × 10^−2^	5
KEGG	KEGG:05144	Malaria	1.69 × 10^−2^	2

Abbreviations: BP, Biological process; CC, Cellular component; GO, Gene ontology; KEGG, Kyoto Encyclopaedia of Genes and Genomes; MF, Molecular function.

### 3.4 Hub gene verification and co‐expression analysis

**FIGURE 5:**
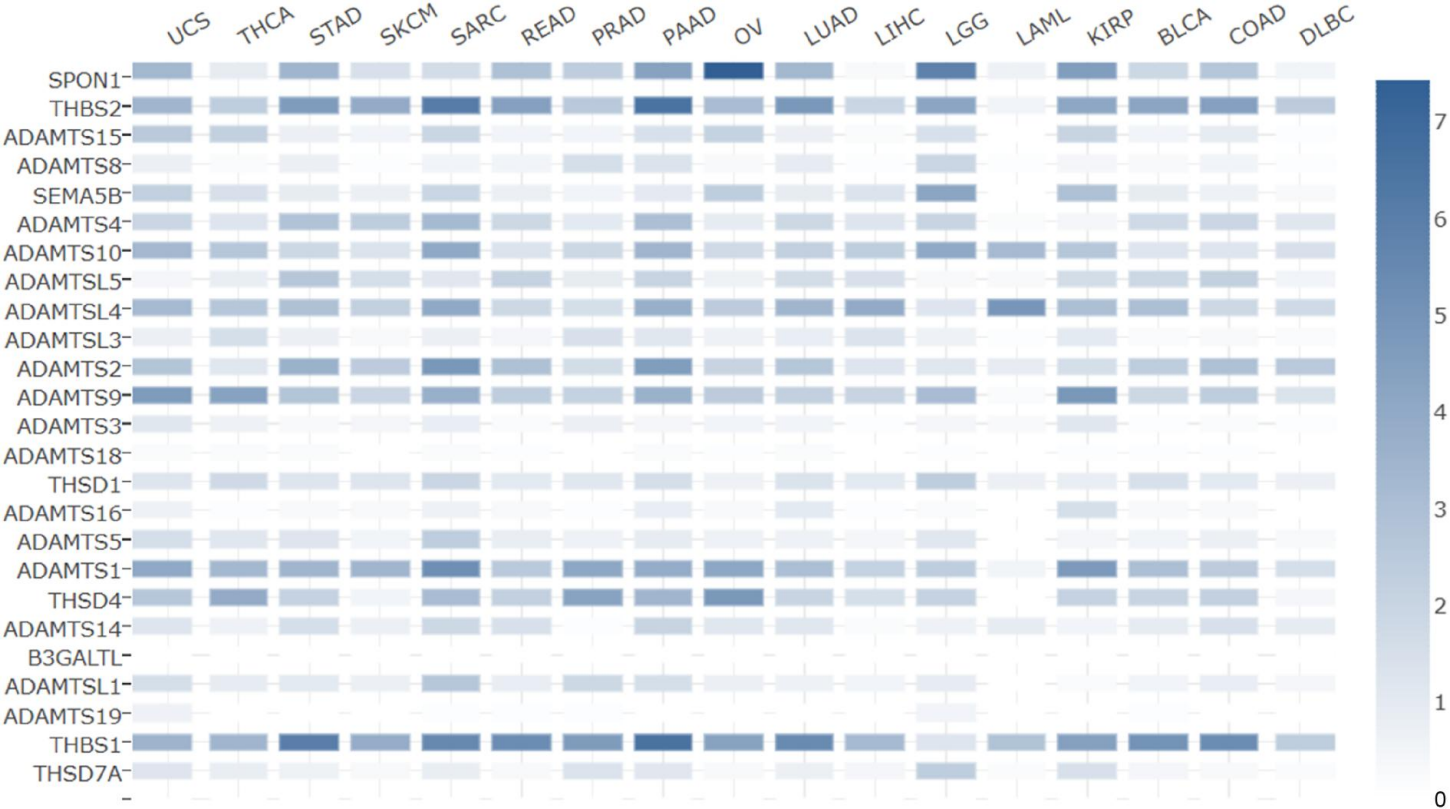
Hub gene expression in multiple human cancers. Deeper colours represent the overexpression of the respective genes. The cancer type abbreviations can be found here: https://gdc.cancer.gov/resources‐tcga‐users/tcga‐code‐tables/tcga‐study‐abbreviations.

We verified the hub genes using GEPIA2 with data from the TCGA and GTEx projects. Figure 5 depicts the levels of expression of the individual genes within the various human cancer types. Among the cancer types, Acute Myeloid Leukemia and Diffuse Large B‐Cell (DLBC) lymphoma were the closest to BL, and in both cases, there was a relatively low expression of the hub genes.

**FIGURE 6:**
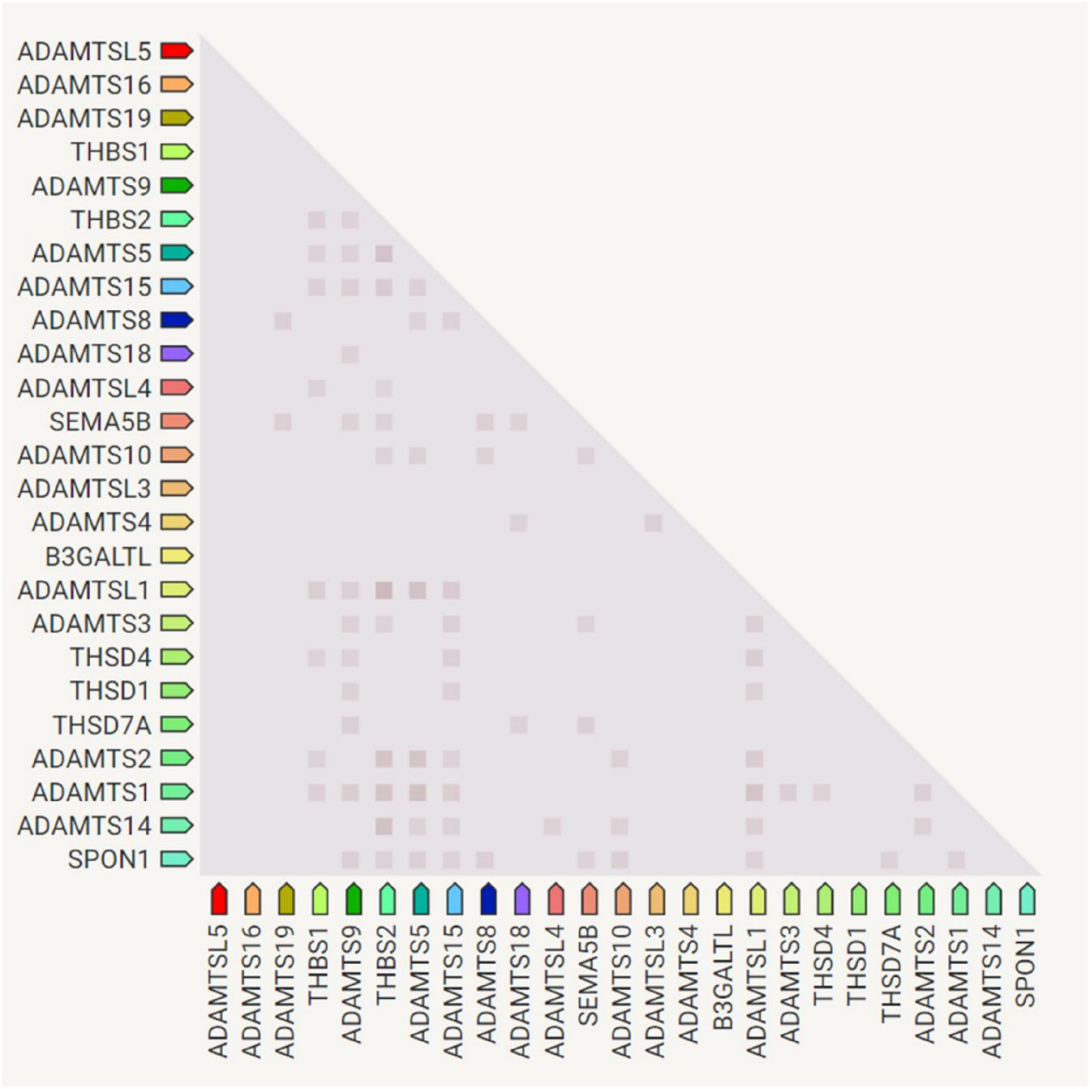
Co‐expression analysis of the 25 hub genes. The deeper the colour, the stronger the association.

Gene co‐expression analysis with STRING also revealed the interactions between the hub genes (Figure 6). RNA expression patterns and protein co‐expression values from the ProteomeHD database were utilised to construct the confidence scores used to generate the relationships. Figure 6 shows that more than half of the genes positively interact.

### 3.5 Hub gene survival analyses

**FIGURE 7:**
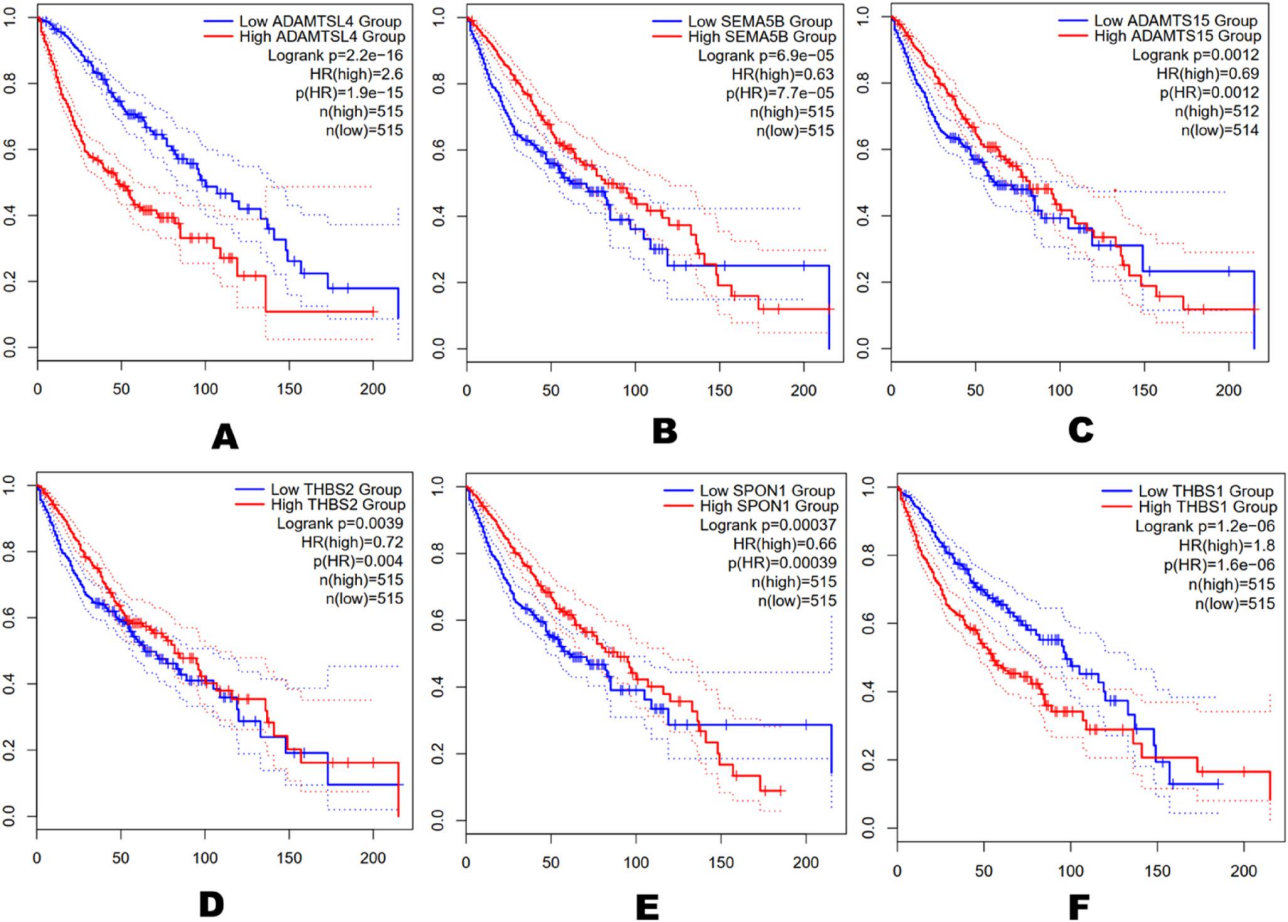
Kaplan–Meier overall survival (OS) analysis of the most significant hub genes. The *x* and *y* axes represent the time (in months) and percent survival, respectively. (a) *ADAMTSL4*, (b) *SEMA5B*, (c) *ADAMTS15*, (d) *THBS2*, (e) *SPON1* and (f) *THBS1*.

The Kaplan‐Meier plotter in GEPIA2 was used to assess the predictive significance of 25 hub genes in the PPI network. The high and low expression of each gene was used to evaluate the overall survival (OS) of BL patients. From Figure 7, the overexpression of *ADAMTSL4* and *THBS1* was negatively associated with OS in BL patients. Low expression of *SEMA5B*, *ADAMTS15*, *THBS2* and *SPON1* was associated with a better OS in BL patients. The rest of the 25 hub genes had no significant correlation (adjusted *p* > 0.05) in patients with BL.

## 4 DISCUSSION

BL is one of the leading causes of facial disfigurations and deaths among children in sub‐Saharan Africa and Northeastern Asia. BL has substantial socio‐economic implications for the patients, their families, and the society. Although the genes implicated in the progression of BL have been extensively explored, research on specific prognostic, diagnostic and therapeutic markers for BL is limited. This study is novel, as it explores common biomarkers that could be used in the management of BL across Africa. In the present study, we pooled and analysed RNA‐seq data from several African BL sequencing projects. Our objective was to delineate a common gene expression pattern for BL patients across Africa and explore genes that can be diagnostic and prognostic markers for BL management.

We ran the data through a standard RNA‐seq analyses pipeline, using DESeq2 as the DEA tool. We found 6314 genes to be significantly differentially expressed (adjusted *p*‐value less than 0.05 and fold change of ±2). The downregulated genes were 4291, whereas 2023 were upregulated. Gene and pathway enrichment analyses showed that the DEGs were primarily implicated in tube development, signalling receptor binding, viral protein interaction, protein‐protein digestion and cell migration, response to external stimulus, serine hydrolase activity and PI3K‐Akt signalling pathway. Response to external stimulus suggests that the organism faces a condition atypical of the normal functioning of the tissues or organs, and it is directly linked to the occurrence and progression of numerous human diseases [19].

Deregulation of the PI3K‐Akt signalling pathway, cell migration and serine hydrolase activity has been reported as critical in cancer development [20, 21]. It has been established the role of serine hydrolases in multiple physiological processes in humans, including cancer [22], oxidative stress [23], metabolism [24] and bacterial infection [25]. Shields et al. [26] unravelled the retinoblastoma‐binding protein 9 (*RBBP9*) as a tumour‐associated serine hydrolase upregulated in pancreatic carcinomas patients. *RBBP9* suppresses the activity of TGF‐β, which is implicated in carcinogenesis [27]. TGF‐β acts as a tumour suppressor by promoting apoptosis and inhibiting cell cycle progression [27].

We further investigated the interactions between the differentially expressed genes by constructing a PPI network. Attention was paid to the network module with the highest connectivity degree among all the resulting 78 modules from MCODE. This module contained 25 genes and 299 interactions, with a score of 24.92. GO and KEGG pathway analyses revealed the 25 genes to be enriched in metalloendopeptidase activity, metallopeptidase activity, hydrolase activity and Extracellular matrix organisation. Cheng et al. [28] underscored membrane metalloendopeptidase (MME) as an essential player in prostate cancer (PC) development. MME works in tandem with *PTEN*, a tumour suppressor, to control the activities of prostate progenitor cells and ultimately suppress PC progression [28]. Li et al. [29] also delineated the functions of MME in oesophageal squamous cell carcinoma progression. The roles of peptidases in tumourigenesis have been extensively studied by Young et al. [30] and Arrebola et al. [31].

The survival analyses of the 25 hub genes were performed using GEPIA2, and the genes that gave statistically significant results were *ADAMTSL4*, *SEMA5B*, *ADAMTS15*, *THBS2*, *SPON1* and *THBS1*. F‐spondin 1(*SPON1*) forms a significant component of the extracellular matrix protein and promotes nerve precursor differentiation [32]. *SPON1* also promotes the growth and development of axons in the peripheral nervous system and spinal cord [32]. Previous studies have shown that overexpression of *SPON1* was linked to poor OS in bladder cancer patients [33], GC cohort [34] and in a PC cohort [35]. In hepatocellular carcinoma, microRNA‐506 binds to *SPON1* to inhibit cell proliferation, migration and invasion [36]. *SPON1* was found to be downregulated in colorectal cancers (CRC) compared to healthy colorectal tissues, which was associated with a better OS in CRC patients. *SPON1* was found to be downregulated in DLBC and upregulated in Bladder Urothelial Carcinoma. However, the exact role of *SPON1* in the development of BL remains to be explored.

The transmembrane protein Semaphorin‐5B (*SEMA5B*) belongs to a family of Semaphorins, which are known to play significant roles in axon growth and maturation during the development of the nervous system [37]. It regulates tumour growth and metastasis, as well as bone metastases and microvascular disorders [38]. *SEMA5B* has been demonstrated to contribute to renal cell carcinoma (RCC) progression [39]. The overexpression of *SEMA5B* has been linked to the development and proliferation of RCC cells and poor prognosis in gastric adenocarcinoma [40]. It has been proposed that Mitogen‐Activated Protein Kinases signalling, notch signalling and tumour signalling are the likely pathways regulated by *SEMA5B* in GC [40].

The thrombospondin 1 (*THBS1*) acts within the tumour microenvironment to stimulate tumour cell motility, regulates antitumour immunity, inhibits angiogenesis, control tumour growth factors and extracellular proteases [41]. Although THBS1 is not usually mutated in most cancers, its expression is regulated by multiple tumour suppressor genes and oncogenes, making it a major carcinogenic player [41]. The implication of *THBS1* in human cancers is context‐dependent and has been reported in gastric carcinomas, bladder and CRC.

Zhang et al. [42] linked the overexpression of *THBS1* to chemotherapy resistance and overall poor prognosis in patients with GC. Prior studies have demonstrated that overexpression of *THBS1* and *THBS2* leads to poor OS in GC patients and has the potential to act as both diagnostic and prognostic biomarkers for GC management [43]. These findings were consistent with Deng et al.'s [44] studies in another GC cohort.

Additionally, Berger et al. [45] reported *THBS2* as a potential biomarker for managing pancreatic cancers. These studies provide evidence for the implication of THBSs in multiple human cancers. However, multiple independent studies have associated decreased expression of *THBS1* with poor prognosis in several cancers, including GC [46], non‐small cell lung carcinoma [47] and oral squamous cell carcinomas [48]. Taken together, the expression of the THBSs in human cancers is context‐dependent and requires further research in other cancer types.

ADAMTSs (A Disintegrin and Metalloproteinase with Thrombospondin motifs) family of glycoproteins have been reported in multiple BP, including angiogenesis, cell migration, carcinogenesis, arthritis and coagulation [49]. Zhao et al. [50] showed *ADAMTSL4* to be directly linked to poor prognosis in patients with Glioblastoma Multiforme and the expression of *ADAMTSL4* to be correlated with immune responses such as infiltration of the tumour by immune cells. Deregulation of *ADAMTSL4* has also been reported in nasopharyngeal carcinoma [51], acute lymphoblastic leukaemia [52] and oesophageal squamous cell carcinoma [53]. Low expression of *ADAMTS15* was demonstrated to be associated with poor prognosis in breast carcinoma [54]. Moreover, studies by Viloria et al. [55] revealed that elevated expression of *ADAMTS15* restricted the growth, invasion and metastasis of CRC cells. Binder et al. [56] also reported that the overexpression of *ADAMTS15* in PC patients was directly linked with OS and consistent with findings from Molokwu et al. [57]. These independent studies collectively provide evidence that the expression levels of *ADAMTSL4*, *SEMA5B*, *ADAMTS15*, *THBS2*, *SPON1* and *THBS1* have the potential to serve as diagnostic and prognostic targets for the management of BL.

This study is limited by our inability to validate the biomarkers using molecular methods, such as quantitative polymerase chain reaction, Microarray and gene knockout analyses. We propose further research to be conducted to ascertain the functions of these makers in the progression of BL.

## 5 CONCLUSION

By analysing gene expression data from BL transcriptome sequencing projects, six biomarkers (*ADAMTSL4*, *SEMA5B*, *ADAMTS15*, *THBS2*, *SPON1* and *THBS1*) were identified as possible indicators for diagnostic and prognostic targets for BL management. Moreover, these genes were implicated in other human cancers and significantly impacted patients' OS. Further research is warranted in mouse models to elucidate the exact roles of these genes in BL development and management.

## AUTHOR CONTRIBUTIONS

**Albert Doughan**: Formal analysis; Validation; Investigation; Data Curation; Writing – Original Draft; Writing – Review & Editing. **Samson Pandam Salifu**: Conceptualisation; Validation; Resources; Writing – Review & Editing; Supervision; Funding acquisition.

## CONFLICT OF INTEREST

The authors declare that they have no competing interests.

## Data Availability

The data that support the findings of this study are openly available in National Centre for Biotechnology Information Short Read Archive (NCBI‐SRA) under the Accession numbers SRP062178, SRP099346 and SRP009316.
